# Issues Raised Involving the Copper Hypotheses in the Causation of Alzheimer's Disease

**DOI:** 10.4061/2011/537528

**Published:** 2011-09-14

**Authors:** George J. Brewer

**Affiliations:** ^1^Department of Human Genetics, University of Michigan, Ann Arbor, MI 48109, USA; ^2^Department of Internal Medicine, University of Michigan, Ann Arbor, MI 48109, USA; ^3^Adeona Pharmaceuticals, Ann Arbor, MI 48103, USA

## Abstract

I present evidence that the epidemic of Alzheimer's disease is a new phenomenon exploding in the latter part of the 20th century in developed countries. I postulate that a major causative factor in the epidemic is the coincident use of copper plumbing, and the ingestion of inorganic copper leaching from the copper plumbing. I present evidence to support this hypothesis and discuss various objections and criticisms that have been raised about the hypothesis, and my responses to these criticisms. I conclude that the hypothesis is well supported by the evidence and deserves serious consideration, because if it is valid, it indentifies a partially preventable cause of Alzheimer's disease.

## 1. Introduction

Alzheimer's disease (AD), and mild cognitive impairment (MCI) that precedes it, has become a huge medical problem. The numbers are staggering. In the US there are currently about 5 million cases of AD and a similar number of cases of MCI [1]. We are in the US, so we are most familiar with US numbers, but there is a similar high incidence in Europe and most other developed countries of the world. The economic costs are also staggering. These patients live many years after the diagnosis and require increasing health and caregiver care. Perhaps most important, the affected patients are robbed of a reasonable quality of life during their elderly years. As the disease progresses, they gradually become unaware of what is going on around them, fail to recognize their loved ones, and lose the ability to function. This loss of quality of life and dignity in such a large proportion of our elderly should be unacceptable to us as a society if it is at all preventable. We should leave no stone unturned to find preventable causes, if any exist, and to develop new therapies.

We have come up with a hypothesis for a plausible, potentially preventable, cause, namely, ingestion of inorganic copper [2]. We do not claim this as a sole cause, but rather it, along with a high fat diet, sets the stage for the disease to develop, particularly if other risk factors are present.

What do we mean by inorganic copper? We differentiate what we call organic copper, which is copper in food, and which is bound up in organic protein molecules, from simple salts of copper not bound to anything. The latter, inorganic copper, is the kind of copper present in drinking water and in copper supplements present in most available vitamin/mineral supplement pills. Since there is almost no protein in uncontaminated drinking water, copper leached off copper plumbing pipes will combine with the cations found in drinking water, such as sulfates, carbonates, and phosphates, to form copper sulfate, copper carbonate, and copper phosphate. Copper added to vitamin/mineral supplement pills is also a simple salt, often copper sulfate. As I will discuss, I believe inorganic copper, at least in part, is handled differently by the body than organic copper, and ends up in different places.

At this point, I will briefly review some aspects of the metallochemistry of AD related to copper and zinc, so that less specialized readers may have a better understanding of the points being discussed. One of the hallmarks of AD pathology in the brain is extracellular amyloid plaques [3]. These are polymers of beta amyloid, a polypeptide clipped off the end of the amyloid precursor protein. The amyloid plaques are thought to be toxic to neurons and are thought to be an integral part of the pathogenesis of AD. These plaques are very rich in copper and zinc, and for a time it was thought both elements were involved in plaque formation [4, 5]. However, it now seems likely that copper is required for plaque formation while zinc is simply bound in large amounts to the plaques. Indeed the plaques, representing a sink for zinc, may add harmful depletion of zinc in the brain [6]. It is already known, based on serum zinc levels, that AD patients are zinc deficient [7], and zinc plays important roles in neuronal function. Thus, copper may be incriminated as a toxic agent, while zinc may be protective, and the brain zinc deficient. Copper also contributes to generation of toxic oxygen radicals in interaction with amyloid plaques [8]. These roles make extracellular copper a potential culprit in the pathogenesis of AD.

Adding further fuel to the fire of a role for copper in AD pathogenesis is a series of papers from an Italian group led by Dr. Rosanne Squitti. This group has focused on “free copper” in the blood. Free copper is the copper in the blood not covalently bound to ceruloplasmin, a copper containing protein in the blood that accounts for 60–65% of blood copper. The remaining copper is called free copper, although it is not really free, but is loosely bound to molecules such as serum albumin. Although not technically “free”, this copper is freely available to contribute to cellular needs, and if the free copper pool is expanded, to cause toxicity. This is most markedly seen in untreated Wilson's disease, an inherited disease of copper accumulation and copper toxicity [9]. The greatly expanded free copper pool in this disease is associated with copper toxicity in brain and liver. Squitti and colleagues have found that the free copper pool in the blood is also increased in AD patients [10], that the increase in free copper is correlated with a cognitive measure [11] and is predictive of cognitive decline over the next year [12]. Although these data do not prove copper causation, they are strong pieces of evidence consistent with the copper causation theory.

In 2003, Sparks and Schreurs [13] did a landmark study that strongly indicated that trace amounts of copper in drinking water are toxic to the brain. They showed that addition of 0.12 ppm copper to the drinking water of a rabbit model of AD greatly enhanced the amyloid plaque pathology in the rabbit brain, but also caused a strong deterioration in cognition. This work has been replicated in other AD models [14], and by other workers [15]. For reference, the EPA allows 1.3 ppm copper in US drinking water for humans, over 10-times the level enhancing AD in the animal models. For further reference, if this amount of copper were increased in food, it would have a trivial, nontoxic effect. The human diet ranges from about 0.8 mg to 1.4 mg/day of copper content. 0.12 ppm additional copper would add 0.12 mg/day, about 10% of the copper already there, and much less than the 50% plus variation in copper already present in the diets. These data indicate that copper from drinking water is much more toxic to the brain than copper from food. I differentiate these two types of copper by referring to copper in drinking water as inorganic copper, as opposed to the copper in food, which is bound to food protein, which I call organic copper. Since there are no significant amounts of protein in uncontaminated drinking water, by the above definition copper in drinking water has to be inorganic.

Although not specifically focused on AD, there is another study which speaks to the potential brain toxicity of inorganic copper. Morris and colleagues [16] did a large study in Chicago in which they evaluated intake of various nutrients and looked at cognition change over a several-year period. They found that subjects in the highest quintile of copper intake, if they also ate a high fat diet, lost cognition at six-times the rate of other groups. Subjects were in the highest quintile of copper intake by virtue of daily ingestion of vitamin-mineral supplement pills containing 1–3 mg of copper. This copper is inorganic by our definition, because there is no protein in these supplement pills.

In a very nice review of whether there is too much or too little copper in Alzheimer's disease, Quinn et al. [17] reviews the evidence for both sides. He keeps an open mind but seems to favor two clinical trial approaches. One is copper lowering, with agents like tetrathiomolybdate or zinc, using ceruloplasmin levels as a guide to copper status. The second is a copper redistribution approach in which the drug, such as one called PBT2, may redistribute copper from extracellular to intracellular compartments. This approach envisions an intracellular copper deficiency being present.

Returning to our inorganic copper causation hypothesis for AD, as we have put this hypothesis forward in various publications, presentations, and manuscript submissions, we have run into a blizzard of objections, counter arguments, and denial. These have come from publications, from reviewers of our papers, from communications with workers in the field, and Alzheimer's physicians. While we expect a good deal of skepticism because our ideas about inorganic copper as partially causative are quite different from what has been generally believed, we are a bit taken back by some of the arguments against our hypothesis being definitely and provably wrong, and others more or less obviously wrong. In some cases there is obvious conflict of interest, with objections coming from the copper industry and those funded by the copper industry. In other cases there is probably bias against a new idea because people have become comfortable with their own existing ideas. In other cases there may be discomfort with recognizing a major harmful factor in our environment that perhaps should have been recognized some time ago, if not long ago.

Thus, I have put this paper together to list the set of subhypotheses that lead to our overarching hypothesis, and to discuss the issues that have been raised surrounding each. I acknowledge the fact that ingestion of inorganic copper as a partially causative factor in AD is still an incompletely proven hypothesis, but I point out there is compelling evidence to support it. So, while my concepts are still an unproven theory, I hope our skeptics will keep an open mind and examine the evidence. Even if someone views our theories as only having a slight chance of being correct, if they do prove correct, it is extremely important for AD prevention. So let us get on with the debate, and the further work needed to see if we can eliminate a preventable cause of AD.

## 2. The Copper Causation Hypothesis

**Overarching Hypothesis**

Ingestion of inorganic copper from drinking water and from copper supplements is a risk factor for AD and a major factor causing the AD epidemic.

**Subhypotheses**

1. The epidemic of AD is a new disease phenomenon and is associated with development.
2. Ingestion of inorganic copper leached from copper plumbing is a major factor in causing the AD epidemic, with copper supplements possibly playing a subsidiary role.
3. Ingestion of inorganic copper contributes directly to the free copper pool in the blood.
4. Elevated free copper in the blood of AD patients is a pathogenic factor in AD.

**(1) Subhypothesis 1: The Epidemic of AD Is a New Disease Phenomenon and Is Associated with Development**

The relationship of this subhypothesis to copper is that in subhypothesis 2 I am going to postulate that the epidemic of AD is associated with the explosive use of copper plumbing in the 20th century, and that copper leaching from the copper pipes is partially causal of AD. Thus, I wish to establish that the epidemic of AD represents something new, not present, or hardly present, in the 19th century, for example, and is associated with use of copper plumbing in the 20th century.

**Supporting Data**

Regarding the question of whether the AD epidemic is a new disease phenomenon, the book by Waldman and Lamb [18] is very well researched on this topic. As far as I know, these are the first authors to suggest the AD epidemic is a new disease phenomenon, and if so, deserve credit for this concept. They reviewed the extensive writings of disease authorities who published in the latter part of the 19th century, and found, consistently, that AD did not exist, or if it did, it must have been rare. There is the writing of Osler, an internist, who compiled a seven-volume series of books, with the help of colleagues, on all medical diseases. Volume 7, almost 1000 pages long [19], was on diseases of the brain and nervous system. There is no mention of an AD-like disease. Similarly, Gowers, a neurologist, wrote extensively on brain and neurologic diseases, and did not mention an AD-like disease [20]. Similarly Freud (work collected by Strachey et al.) who wrote extensively on psychiatric disease, did not describe an AD-like disease [21]. Finally Boyd, who wrote a comprehensive textbook on pathology, which was put out several times as updated new editions, did not describe the hallmarks of AD pathology in the brain, amyloid plaques, and neurofibrillary tangles [22].

Since AD is a disease of aging, Waldman and Lamb [18] wondered whether lack of elderly people in the population when Osler, Gower, Freud, and Boyd made their observations was the cause of the absence of the disease in their writings. To the contrary, they found that there was always a substantial fraction of people over 60 in the relevant populations. For example, in France, in 1911, more than half of the population was still alive at age 60.

I have examined the US census figures for 1900 and found that there were 3,184,363 people over age 60. At today's prevalence, there should have been over 36,000 US cases, more than enough for Boyd to find positive AD brains at autopsy.

Regarding the question of whether the AD epidemic is largely associated with development, Ferri et al. [23] give a summary of data for developed and undeveloped countries. In general the data show a considerably higher frequency of AD in developed countries.

**Contrary Data, Contrary View, and My Responses:**

**Contrary View**

A common view among AD workers is that AD was always present, but the dementia was simply passed over as part of normal aging, and no special attention was paid to these patients.

**My Response**

This might account for the lack of attention by the clinicians (Osler [19], Gowers [20], and Freud [21]), although it is a little hard to believe given their quality and attention to detail, but it does not explain why the pathologist Boyd [22] did not see amyloid plaques and neurofibrillary tangles, pathological hallmarks of AD, in the brains of patients autopsied.

**Contrary View**

Another common view is that the AD incidence is increasing only because the disease is now better recognized and labeled, and because of the increasing number of elderly people in the population.

**My Response**

I agree that both of these factors are involved in explaining the increasing prevalence. However, my position is that there has to be something additional to explain the absence of brains with AD pathology around the beginning of the 20th century [22], and the very high current frequency of such brains at autopsy. We pointed out in the previous section that an elderly population was present in France and the US 100 years ago, so simply more aged people in the current population cannot explain the almost total absence of the disease back then.

**Contrary View**

Another view is that the high fat, high calorie diets of developed countries are a major causative factor and a major reason for the explosive epidemic of AD in the Western world. Indeed, Grant [24] has shown a strong positive correlation between fat intake and AD prevalence across numerous countries.

**My Response**

That a high fat diet is a major causative factor for AD is a reasonable hypothesis. My hypotheses is that a high fat diet interacts with copper to contribute to causation. Thus, the Sparks and Schreurs [13] study involved cholesterol feeding in the rabbit model where trace amounts of copper greatly enhanced the disease, and the Morris et al. [16] study found that those in the highest quintile of copper intake, also eating a high fat diet, had the greatest decline in cognition. There are biochemical reasons why the two factors might interact in causation, for example, copper oxidizes cholesterol and other fats into substance toxic to the brain. However, a major role for copper whether in the presence of a high fat diet or not, is supported by trace copper enhancement of AD-like disease, in the mouse model of AD, which does not receive a fat- or cholesterol-enriched diet [14, 15]. Thus, my current view is that both factors are causative, and interact together to further enhance causation.

**(2) Subhypothesis 2: Ingestion of Inorganic Copper Leached from Copper Plumbing Is a Major Factor in Causing the AD Epidemic with Copper Supplements Possibly Playing a Subsidiary Role**

**Supporting Data**

To give the reader insight as to possible involvement of copper in AD causation, I would like to briefly review the interaction of copper with the molecules intimately involved in AD pathogenesis. Beta amyloid, the key molecule in amyloid plaques, binds copper and cholesterol, causing oxidation of cholesterol to 7-OH cholesterol, extremely toxic to neurons [25, 26]. Beta amyloid is cleaved from amyloid precursor protein by beta secretase. Amyloid precursor protein binds copper, and reduces it from copper (II) to copper (I), which produces oxidative damage [27, 28]. Beta secretase also binds copper for activity. Both amyloid plaques and neurofibrillary tangles, hallmarks of AD brain pathology, are major sites of generation of toxic oxygen radicals [29]. Deferoxamine or EDTA, an iron or general metal chelator, respectively, abolishes the generation of these radicals, but replenishment with iron or copper restores the activity [29]. Tau protein, a major building block for neurofibrillary tangles, binds copper, which is a causative factor in tau aggregation [30].

In response to the six contrary views later in this section, I will show how AD risk factors, apolipoprotein E4, homocysteine, levels, and certain transferrin alleles, all tie in with the copper causation hypothesis.

A major initiating stimulus for all of our thinking and leading to the hypotheses proposed here was the 2003 study of a cholesterol-fed rabbit model of AD by Sparks and Schreurs [13]. By accident, but also by serendipity, they discovered that trace amounts of copper (0.12 ppm) added to the otherwise distilled drinking water of these rabbits, greatly enhanced the AD-like pathology in the brains, and greatly decreased the rabbits' ability to perform previously learned tasks. In other words, trace amounts of copper in the drinking water caused the two hallmarks of AD, amyloid plaques and loss of cognition, to worsen. For reference, the US Environmental Protection Agency (EPA) allows up to 1.3 ppm copper in human drinking water, over 10-times as much as worsened AD in the rabbit model. Similar and even higher, levels of copper are permitted in drinking water around the world. Sparks et al. [14] later confirmed the AD enhancing effects of trace amounts of copper in noncholesterol-fed AD models, such as in the mouse model. The effect of these very low amounts of copper added to drinking water enhancing AD pathology was confirmed by a group from Rochester [15].

There is a rough correlation between the use of copper plumbing tube around the world and the incidence of AD. This is consistent with the hypothesis that copper leaching from copper plumbing is causing AD.

Are there any data on whether significant amounts of copper are actually present in the drinking water of a developed country, such as the U.S? Fortunately, there is. As a result of evaluating copper levels in drinking water of my Wilson's disease patients, I accumulated data on 280 samples of home drinking water from households all across North America. The data are shown in Table 1. The data show that 2% of samples are over the EPA limit of 1.3 ppm, 31% are over 0.12 ppm, the level that enhanced AD in the rabbit and other animal models, 41% are between 0.12 ppm and 0.01 ppm, a level of unknown toxicity/safety, and only 28% are less than 0.01 ppm, a level we consider safe. Thus, 72% of these North American drinking water samples either have copper levels above that enhancing AD in animal models, or are high enough to be of unknown safety in that regard.

The other common source of inorganic copper ingested by humans is copper in vitamin/mineral supplements. In the last 50 years or so it has become very common in the US, and probably many other developed countries to ingest one or more of these supplement pills per day. Almost all of these pills have 1 to 3 mg of copper. Since the dietary intake of copper is about 1.0 mg per day [31–34], this is a hefty dose of copper, and it is all inorganic. Is there any evidence that this high dose of inorganic copper has an effect on cognition? Yes, in a large study of a general Chicago population published in 2006, Morris and colleagues [16] found that those in the highest quintile of copper intake, if they also ate a high fat diet, lost cognition at six-times the rate of the rest of the people. These people were in the highest quintile of copper intake because they took mineral supplement pills containing copper.

**Contrary Data, Contrary Views, and My Responses**

Recently I wrote a letter to the editor criticizing a collection of five papers published in one issue of the Journal of Toxicology and Environmental Health [35]. These papers concerned the safety and toxicity of oral copper intake. In general, these papers were nice reviews of various aspects of the topic. My two main criticisms in my letter to the editor were that none of the five papers differentiated the toxicity of inorganic copper in drinking water or in copper supplements from the toxicity of copper bound to organic molecules in food, and none of them referred to the literature emerging since 2003 on the toxicity of this inorganic copper. There was a lengthy response senior authored by Danzeisen et al. [36] to my letter by 8 of the 14 authors of these five papers plus four other scientists who were recruited to join in on the discussion. In spite of the length of this response, it did not address the two main criticisms listed above. But it did criticize many aspects of the data cited in my letter that pointed to the potential toxicity of inorganic copper ingestion. These criticisms as they relate to the inorganic copper causation of AD hypothesis will be discussed below.

**Contrary View**

One of the criticisms in the Danzeisen et al. [36] paper was leveled at the Sparks and Schreurs [13] rabbit model study. The authors mistakenly asserted that the study only showed amyloid plaque reduction in the animals, and that plaque reduction may not be related to “progression of dementia.”

**My Response**

Danzeisen et al. [36] are mistaken. The Sparks and Schreurs [13] paper showed not only plaque reduction but loss of cognition in the copper treated animals.

**Contrary View**

The Danzeisen et al. [36] paper criticizes Brewer et al.'s [35] conclusion from the Sparks and Schreurs [13] study that copper exposure from drinking water in itself can be a risk factor for AD. Danzeisen et al. [36] are concerned about drawing conclusions from a rabbit model of AD in which high cholesterol feeding preconditions the animal to AD, so that worsening of the disease from addition of copper to drinking water cannot be used to conclude that copper alone in drinking water is a risk factor for AD. To quote them, “This type of study in compromised animals is applicable to neither the derivation of the maximum contaminant level (MCL) for copper in drinking water, nor its extrapolation to the nonobserved-adverse-effect level (NOAEL) for copper in humans.”

**My Response**

In my opinion drinking water should be nontoxic and safe for all segments of the population. When I served on the Committee to Evaluate Copper in Drinking Water convened by the National Research Council in 2000 [37], we considered, for example, whether the current allowable level of copper was safe for Wilson's disease heterozygous carriers, who have minor extra accumulation of copper, and comprise only 1% of the population. Similarly, if the segment of the population who eat a high cholesterol, high fat diet, which is increasingly common in our society, are especially vulnerable to get AD if they ingest low levels of copper in drinking water, we must try to make the water safe for them. And I point out that the mouse AD model, which also showed vulnerability to 0.12 ppm copper in drinking water, does not depend on cholesterol feeding to produce the disease [14].

I believe we have to be more comprehensive in assessing the safe limits of copper in drinking water. The standard methods consist of assessing acute effects of copper levels in drinking water in humans, but chronic effects of copper toxicity are evaluated using animal studies of copper in food. The latter is inadequate, because the evidence seems pretty clear from the AD animal model work that copper is much more toxic in water than it is in food. If it takes special animal model studies to reveal potential vulnerability of humans to copper in drinking water for a specific disease, we should pay attention to those studies, not ignore them by saying the animals were “compromised” to produce the model.

**Contrary View**

Another one of the criticisms in the Danzeisen et al. [36] paper was the Brewer et al. [35] citation of the work of Morris et al. [16] referred to earlier. In this work Morris et al. [16] found that those in the highest quintile of copper uptake, most being in this quintile because of taking copper supplements, if they also ate a high fat diet, suffered cognition loss at six-times the rate of other groups, Danzeisen et al. [36] state, “It is not possible to separate the factors studied by these authors and implicate copper directly in the presence of high fat intake.” Danzeisen et al. [36] also worried about separating the effect of copper intake from the other ingredients of vitamin/mineral supplements.

**My Response**

Regarding the criticism of copper being toxic only in the presence of a high fat intake, we point out that these were normal people eating their normal diet. Surely those eating this kind of diet which is an increasing number of our people, also need protection from toxicity resulting from ingestion of inorganic copper in supplement pills that singles them out for cognition loss. The FDA, needs to evaluate the potential toxicity of copper in supplements. Regarding sorting out the effects of copper versus other ingredients in the supplements, Morris et al. [16] did that in their statistical analysis. Copper, plus a high fat diet, were the only culprits.

**Contrary View**

Danzeisen et al. [36] contradict the Brewer et al. [35] statement that, “No formal toxicology studies of copper in drinking water have ever been done,” by directly saying this “is not correct.”

**My Response**

Danzeisen et al. [36] never specifically refute the Brewer et al. [35] statement. Instead they cite various types of data which are not relevant to the point, including an assessment, not based on a chronic toxicity study of copper in drinking water, by the European Union, that up to 2.0 mg/L (2.0 ppm) in water is safe, with the average intake currently being 0.7 ppm.

**Contrary View**

Danzeisen et al [36] cite studies by Kessler et al. [38] in which AD patients were given 8 mg of copper as copper orotate/day for one year. The cognition of the patients did not get worse, leading to the conclusion that long-term oral intake of copper is not a risk factor for AD.

**My Response**

These patients did not improve cognition either! The hypothesis underlying the study was that AD patients were copper deficient, and copper therapy would be efficacious. Thus, the study was a negative one in terms of its underlying hypothesis. However, because the patients did not get worse, the authors turned their conclusion around, and concluded copper administration was not harmful.

As to why the patients did not get worse given our hypothesis about the role of copper toxicity, there are problems with this study leading to many possible explanations. Copper orotate is relatively insoluble, and perhaps very little was absorbed. No copper parameters were measured in the patients, so there is no information on absorption. It is possible that maximum effects from exogenous copper were already occurring, and that more exogenous copper therefore had no additional effect. Finally, AD is a slowly progressive disease, and these were mild patients. Perhaps the observation period was not long enough to affect the cognitive tests being carried out.

**Contrary View**

Danzeisen et al. [36] criticize Brewer et al.'s hypothesis that copper plumbing is a causal factor in AD. They say genetic factors play a “particularly important role” in AD, with age being the greatest nongenetic risk factor. They say “we are not aware of any peer-reviewed scientific evidence that supports Brewer et al.'s [35] conjecture that the use of copper piping is a risk factor in AD.”

**My Response**

I agree that there are genetic risk factors such as the apolipoprotein E4 alleles [39] and certain genes involved in controlling iron, such as certain hemochromatosis alleles [40] and certain transferrin alleles [41]. In addition, elevated homocysteine levels are a risk factor [42]. Interestingly, these all tie in with our hypothesis. The apo E4 protein lacks a copper binding cysteine, so it cannot bind copper while the apo E3 and E2 alleles have this cysteine. Thus apo E4 is unable to remove copper from the brain. Homocysteine interacts with copper to oxidize cholesterol, damaging to neurons [43]. Iron build up increases oxidant stress, as does copper build up, so the two work in the same manner. It is our view that copper toxicity and a high fat diet [24] set the stage for AD to develop, and other risk factors operate on that stage to increase the likelihood of AD to develop. Regarding the comment about no peer-reviewed evidence (in other words no published paper) exists to support our hypothesis about copper plumbing as a risk factor for AD, the explanation is that we claim priority as the first to propose this hypothesis. There has to be a first worker to propose a new hypothesis. If the hypothesis is correct, supporting papers will come later.

**Contrary View**

Danzeisen et al. [36] cite a paper by Crouch et al. [44] which presents evidence that there is an intracellular copper deficiency in the brain of an AD mouse model. They interpret this to mean that ingestion of additional inorganic copper would be a helpful rather than harmful. They also cite papers which they say supports the idea of an overall low level of copper in the human AD brain.

**My Response**

I do not agree that it is clear that the AD brain has a low copper value. There is just as many papers that find copper levels elevated or normal in the AD brain as find it low. So this area is controversial. However, the paper by Crouch et al. [44] is interesting. These authors administer a copper binding agent to AD model mice. The agent is designed to release its copper in the intracellular environment. This agent improved AD-type pathology and improved cognitive performance in a mouse model. A sister compound which bound copper but did not release it intracellularly had no effect. Similar claims for the drug clioquinol are not as clear, because it is not certain that clioquinol releases bound copper intracellularly, and clioquinol also binds and transports zinc.

The data in the Crouch et al. [44] paper do suggest that increasing the intracellular copper levels in the mouse AD model is beneficial. This possibly indicates that there is intracellular copper deficiency. However, there appears to be overlapping functions of copper and zinc in the synapse, and recently it has become clear that AD patients are zinc deficient [7]. It is possible that the effect of copper in the Crouch et al. [44] study is substituting for a relative lack of intracellular zinc.

However, even if an intracellular copper deficiency exists, there is also evidence that extracellular copper enhances oxidant damage in interaction with amyloid plaques and A beta amyloid oligomers. If there is an intracellular copper (and/or zinc) deficiency, and an extracellular copper toxicity, then therapy can be aimed at both, restoring intracellular copper (and/or zinc) levels, and reducing extracellular copper levels. If intracellular copper deficiency exists, it does not negate our hypothesis that excess ingestion of inorganic copper contributes to AD development because of extracellular copper toxicity.

**Contrary View**

It has been suggested that the source water, rather than copper plumbing, may contribute the toxic copper to drinking water.

**My Response**

This may be true in parts of the world, and in a few areas of the US. But in many areas of the US, source water has low copper.

**Contrary View**

The epidemiologic data showing a low rate of AD in Japan [45], a developed country, that shuns copper plumbing, which we use to support the copper plumbing causal hypothesis, has been criticized, because Japanese have a diet different than other countries. We have pointed out that when Japanese migrate to Hawaii, where copper plumbing is common, they develop a rate of AD typical of developed countries [46], in further support of our hypothesis. This concept has been criticized because the diet and other environmental factors may also be different in Hawaii.

**My Response**

We agree the epidemogic data, including the Japanese epidemiologic data, do not prove our hypothesis. The data, including the somewhat unusual Japanese data, simply show consistency with the hypothesis.

**Subhypothesis 3: Ingestion of Inorganic Copper Contributes Directly to the Free Copper Pool in the Blood**

**Supporting Data**

Normally food copper is metabolized by the liver and channeled into safe pathways such as being incorporated in to ceruloplasmin. The exceptional toxicity of trace amounts of inorganic copper in drinking water in AD animal model studies [13–15] suggests that at least some of this copper must follow a different route. We do have a little direct data that this is indeed true. When we administer inorganic copper orally labeled with ^64^Cu, we see a fraction of the label appear in the blood in 1-2 hours, too soon to be processed by the liver [47]. The amount appearing is 5-6% of the administered label at both the 1- and 2-hour time points, so a guesstimate of the area under the curve, a rough estimate of absorption, is about 15%. We believe this fraction of ^64^Cu-labeled inorganic copper is bypassing the liver, is therefore not incorporated safely into ceruloplasmin, contributes directly to the nonceruloplasmin free copper pool, and represents what is happening to at least a portion of the inorganic copper ingested in drinking water or copper supplements. We hypothesize that this pathway for ingested inorganic copper is a causal factor in AD.

**Contrary View**

One criticism of the above is that Ctr 1 is the major copper transporter in intestinal cells [48], so it does not seem likely that inorganic copper can bypass the liver and enter the free copper serum pool directly.

**My Response**

The animal model AD data [13–15] indicating the toxicity of trace amounts of inorganic copper in drinking water, and our ^64^Cu data [47], indicates something different is happening with at least a portion of ingested inorganic copper. However, I agree that the mechanism by which some of the inorganic copper is differently absorbed is unknown. We can speculate that Ctr 1 may only recognize organically bound copper, and some inorganic copper is absorbed through different channels, such as cation channels. Also, it is possible that inorganic copper is absorbed quickly, as a bolus, and some simply bypasses the liver because of the high level. At this point the mechanism is unknown.

**Contrary View**

Danzeisen et al. [36] criticize our ^64^Cu data by saying that we do not know how much inorganic copper actually bypassed the liver and promptly appeared in the blood because the specific activity of the radioactive copper was not measured. They also say, since it was given with milk, some of it probably became protein bound.

**My Response**

Regarding the first criticism, it represents a misunderstanding of this kind of study. It is not necessary to know the specific activity to know the proportion of the label that was involved, which is what is of interest. Regarding the second comment, some of the label probably did bind to ovalbumin becoming “organic” copper. Thus, if the ^64^Cu had been given in water and remained completely inorganic, the 1- and 2-hour blood values might have been considerably higher.

**Subhypothesis 4: Elevated Free Copper in the Blood of AD Patients Is a Pathogenic Factor in AD**

**Supporting Data**

Elegant data on this point has been published by Squitti and her colleagues [10–12, 49]. They have shown the following.

1. Free copper in the blood is elevated in AD patients [10].
2. Free copper negatively correlates with cognition in AD patients [11].
3. Free copper levels predict the rate of loss of cognition in AD patients [12].

They also showed that free copper negatively correlates with cognition in older normal women [49].

Number (1) above (elevated free copper in AD) has been confirmed [50, 51]. Our groups finds a higher percentage of free copper in AD, and increased defective ceruloplasmin, that is, ceruloplasmin that has lost its enzymatic activity because it has lost some of its copper. We do not know whether this process contributes to a higher free copper. Arnal et al. [51] confirm the higher free copper in AD and also confirm a significant negative correlation between serum free copper and cognition.

**Contrary View**

Danzeisen et al. [36] find fault with the way Squitti et al. [10–12, 49] determined free copper “values arithmetically by measuring Cp-bound copper and subtracting the value from total copper.” They say, “There is a scientific consensus (voiced by seven experts in the field in a peer-reviewed article: Danzeisen et al. [52] that non-Cp copper determined by arithmetic method is not sensitive to copper status, and that its true value is very difficult to measure”.

**My Response**

First, non-Cp copper determined arithmetically is not “difficult to measure.” We have been doing it for years in our Wilson's disease work [9, 31, 53]. One simply determines the Cp level by either an enzymatic or immunologic method, calculates the amount of copper bound to Cp from the known amount of copper bound to each mg of Cp, and subtracts that from total serum copper. Of the two ways of measuring Cp, the enzymatic is probably a little better, because in the immunologic method, a little apo-Cp (without copper), is usually measured, the amount determined by the antibody used. But either way, if the same method is used consistently, it gives a very good estimate of the non-Cp copper and its change over time.

If this methodology is so useless, why is it so elevated in untreated Wilson's disease patients experiencing acute copper toxicity [9]? And why did it change so consistently and predictably in our Wilson's disease therapy studies [9], And, coming back to the data being criticized, why does it correlate so beautifully with cognition measures in AD [11], why does it predict cognition loss in AD [12], and why does it correlate with cognition in elderly women [49]? It is not credible to believe all of this is coincidence or random concurrence? Even the staunchest critics, even if they do not believe non-Cp copper is in the pathologic pathway for AD, have to admit it is at least a good marker of the AD process.

**Contrary View**

I have heard criticism that other authors in the literature have reached opposite conclusions. I have already discussed and criticized the work of the Bayer group [38] who administered copper orotate to AD patients. This same group published a paper that showed a lower total serum copper and ceruloplasmin in AD patients with cerebrospinal diagnostic markers, than AD patients without those markers, suggesting severe patients are copper deficient when compared to less severe patients [54].

**My Response**

This work is flawed in that total serum copper was measured not free copper, and total serum copper will simply reflect Cp levels, since Cp contributes up to 90% of serum copper.

**Contrary View**

Bayer et al. [55] have also published a paper showing that dietary copper supplementation reduces amyloid A beta production in an AD mouse model.

**My Response**

However, these mice are severely copper deficient, and copper supplementation helps them in many ways because of this.

**Contrary View**

Klevay [56] has written a review entitled, “Alzheimer's disease as copper deficiency.” Klevay cites the work of the Bayer group [38, 54, 55] already discussed here in support of his hypothesis.

**My Response**

The title of this review is misguided in the sense that it ignores the evidence for what the syndrome of copper deficiency really is. For example, in recent years the syndrome of copper deficiency has been amply illustrated by excessive use of zinc containing-denture adhesive. The zinc eventually causes severe copper deficiency. These patients develop pancytopenia followed by myelopolyneuropathy, which often leaves them severely neurologically crippled [57, 58]. None of this occurs in AD, so it is not reasonable to suggest that AD is a simple copper deficiency [56].

However, as we have previously discussed, we accept the possibility that AD patients have a neuronal intracellular copper deficiency. But this is very different than what Klevay [56] and the Bayer group [38, 54, 55] have been proposing.

**Contrary View**

An opinion has been expressed that the biliary copper export pathway (the one that is defective in Wilson's disease) will protect against increases in serum free copper.

**My Response**

My view is that this pathway is protective, but not immediately sensitive to mild increases in free copper. Thus, if the free copper levels are increased by increased intake, the export pathway will respond and increase export, but steady state levels of free copper are probably increased.

## 3. My Overview of the Status of These Issues

It seems to me that subhypothesis 1 is not all that controversial. Rather the doubters are just not informed. I urge them to read Dying for a Hamburger by Waldman and Lamb [18]. This is an extremely well-researched book on the topic of the AD epidemic being new, and that elderly people have always existed in our population. Even if one thinks AD was passed off as simply the normal dementia of aging, it is very difficult to explain the absence of amyloid plaques and neurofibrillary tangles in the autopsy specimens of Boyd [22]. I am not saying AD did not exist at all in the 1900s. It probably did. Probably occasionally a constellation of risk factors would come together and produce the disease. What I am saying is it was relatively rare, and that there were enough elderly people present for it to have easily shown up in the autopsy specimens if it were occurring at today's rate. Those who wish to counter this argument would have to either show that there were very few people over age 60, or that the plaques and tangles were actually present in autopsy samples.

There is more controversy about subhypothesis 2, as is evident from the length of the contrary view/My response material regarding that subhypothesis.

One area where a legitimate difference of opinion can exist is whether the Sparks and Schreurs [13] and other AD animal work [14, 15], in which trace amounts (0.12 ppm) of copper in drinking water greatly enhances AD pathology and cognition loss, can be extrapolated to the human. There are four animal models studied, one of them does not involve cholesterol feeding (the mouse), and they are all consistent. Plus the mouse work has been confirmed in another laboratory [15]. So the animal model work is very solid that trace amounts of copper in drinking water are sharply more toxic than copper in food in AD-like causation. But still, they cannot be extrapolated to the human with absolute certainty. My position is that they should be taken seriously as signaling a major potential (and preventable) cause of human AD. Certainly not ignored, as did the five papers in the Journal of Toxicology and Environmental Health [35], and not inaccurately criticised [36].

A legitimate point is that besides the increase of copper plumbing in developed countries, many other environmental changes have occurred, as well as behavioral changes in people. Thus, assuming subhypothesis 1 is correct, any of these factors could be responsible for the AD epidemic. Waldman and Lamb [18], who wrote Dying for a Hamburger, in their otherwise well-researched book previously referred to, postulated that it was beef eating, with AD caused by prions in the beef. Beef eating certainly correlates with development, but there is no evidence AD is a prion disease. However, a factor associated with development is eating more fat in the diet (including that in beef). Grant [24] has shown there is a good correlation between a country's dietary fat consumption and the prevalence of AD. Since much of the animal model data (plus the Morris et al. [16] data) involves feeding cholesterol (or ingesting a high fat diet), we believe copper and cholesterol/fat are synergistic in AD causation. Copper can oxidize cholesterol and certain fats into substances that are toxic in the brain. However, one could postulate that a high fat diet, without copper involvement, is the causative factor. This is a reasonable hypothesis. But of course it ignores all the data involving copper. Other unknown causatives factors associated with development are also possible.

I do not believe the issue of whether adequate testing has been done on the chronic toxicity of copper in drinking water is very controversial. It just has not been done. None of the statements or citations by Danzeisen et al. [36] show that this type of work has ever been done. Besides animal studies, epidemiologic studies on humans could be done, relating disease incidence to levels of substances like copper in drinking water. It is not an easy study, given that many people move around and their source of drinking water varies from time to time.

The possibility of a neuronal intracellular copper deficiency in AD, irrespective of potential toxicity from excess extracellular copper, is a viable hypothesis and supported by some emerging data [44]. This would make rational the use of therapies which deliver copper intracellularly while not adding extracellular copper. However, in the face of all the evidence of extracellular copper toxicity in AD, intracellular copper deficiency does not make rational the use of copper supplementation to AD patients as done by the Bayer group [38]. Nor does it justify a review, such as that of Klevay [56], entitled, “Alzheimer's disease as copper deficiency.”

I do not believe there is much controversy about whether some of the ingested inorganic copper contributes directly to the free copper pool, as proposed in subhypothesis 3. Even Danzeisen et al. [36] referring to my interpretation of our ^64^Cu data as showing that some ingested inorganic copper had bypassed the liver said, “this interpretation appears correct.” Of course, the mechanism by which this occurs is still unknown.

Regarding subhypothesis 4, the elegant work by Squitti and colleagues [10–12] showing elevated serum free copper in AD is not very controversial. It has been confirmed by others [50, 51]. Danzeisen et al. [36] criticize the method, but this criticism seems groundless. What is still to be debated and studied is what is the significance of this elevation? Does a higher serum free copper mean greater penetration of the blood-brain barrier and an elevation of extracellular toxic copper in the brain? Or alternatively, is it just a biomarker of other, critical, pathogenic pathways? It is our hypothesis that it is in the pathogenic sequence.

In conclusion, I believe our overarching hypothesis and subhypotheses, of a key role of inorganic copper from drinking water and supplements in causation of a new epidemic, an epidemic of AD, is well supported. I do not believe any of the contrary views, while some raise valid points to consider, have dealt fatal blow to my hypotheses.

I emphasize that at this point my concepts of copper, as one significant causative factor among others, are not finally proven. I believe they are important because if they are correct, they point to a method of at least partial prevention of AD, by lowering copper levels in drinking water and stopping ingestion of copper supplements. Those that take those steps now may benefit while waiting for final proof, which may take a long time.

## Figures and Tables

**Table 1:** Copper levels in Nor American household drinking water.

Copper level (ppm)	Number of households			
Undetectable 0.01		80 (28%)	12	Considered safe
			68	

0.02		114 (41%)	36	Safety unknown
0.03			16	
0.04			13	
0.05			15	
0.06			12	
0.07			7	
0.08			7	
0.09			8	

0.1		81(29%)	3	Cause Alzheimer's disease in animal models
0.2			34	
0.3			14	
0.4			9	
0.5			6	
0.6			2	
0.7			3	
0.8			2	
0.9			2	
1.0			2	
1.1			2	
0.2			1	
1.3			1	

1.4		5 (1.8%)	1	Above EPA limit
1.8			1	
1.9			1	
3.4			1	
6.0			1	

## References

[1] Alzheimer’s Association

[2] Brewer GJ, Newsome DA (2010). *Toxic Copper: The Newly Discovered Culprit in Alzheimer’s Disease and Dementia*.

[3] Mally W, Caldwell P (1998). *Alzheimer’s Disease*.

[4] Atwood CS, Moir RD, Huang X (1998). Dramatic aggregation of alzheimer by Cu(II) is induced by conditions representing physiological acidosis. *Journal of Biological Chemistry*.

[5] Bush AI, Pettingell WH, Multhaup G (1994). Rapid induction of Alzheimer A*β* amyloid formation by zinc. *Science*.

[6] Adlard PA, Bica L, White AR (2011). Metal ionophore treatment restores dendritic spine density and synaptic protein levels in a mouse model of Alzheimer’s disease. *PLoS ONE*.

[7] Brewer GJ, Kanzer SH, Zimmerman EA (2010). Subclinical zinc deficiency in Alzheimer’s disease and Parkinson’s disease. *American Journal of Alzheimer’s Disease and other Dementias*.

[8] Religa D, Strozyk D, Cherny RA (2006). Elevated cortical zinc in Alzheimer disease. *Neurology*.

[9] Brewer GJ, Askari F, Dick R (2009). Treatment of Wilson’s disease with tetrathiomolybdate: V. Control of free copper by tetrathiomolybdate and a comparison with trientine. *Translational Research*.

[10] Squitti R, Pasqualetti P, Dal Forno G (2005). Excess of serum copper not related to ceruloplasmin in Alzheimer disease. *Neurology*.

[11] Squitti R, Barbati G, Rossi L (2006). Excess of nonceruloplasmin serum copper in AD correlates with MMSE, CSF *β*-amyloid, and h-tau. *Neurology*.

[12] Squitti R, Bressi F, Pasqualetti P (2009). Longitudinal prognostic value of serum "free" copper in patients with Alzheimer disease. *Neurology*.

[13] Sparks DL, Schreurs BG (2003). Trace amounts of copper in water induce *β*-amyloid plaques and learning deficits in a rabbit model of Alzheimer’s disease. *Proceedings of the National Academy of Sciences of the United States of America*.

[14] Sparks DL, Friedland R, Petanceska S (2006). Trace copper levels in the drinking water, but not zinc or aluminum influence CNS Alzheimer-like pathology. *Journal of Nutrition, Health and Aging*.

[15] Deane R, Sagare A, Coma M A novel role for copper: disruption of LRP-dependent brain Abeta clearence.

[16] Morris MC, Evans DA, Tangney CC (2006). Dietary copper and high saturated and trans fat intakes associated with cognitive decline. *Archives of Neurology*.

[17] Quinn JF, Crane S, Harris C, Wadsworth TL (2009). Copper in Alzheimer’s disease: too much or too little?. *Expert Review of Neurotherapeutics*.

[18] Waldman M, Lamb M (2005). *Dying for a Hamburger : Modern Meat Processing and the Epidemic of Alzheimer’s Disease*.

[19] Osler W (1910). *Modern Medicine in Theory and Practice*.

[20] Gowers WR (1888). *A Manual of Diseases of the Nervous System*.

[21] Strachey J, Freud A, Strachey A, Tyson A (1966). *24 Volumes Entitled, The Standard Edition of the Complete Psychological Works of Sigmund Freud, Written between 1895 and 1939*.

[22] Boyd W (1938). *A Textbook of Pathology: An Introduction to Medicine*.

[23] Ferri CP, Prince M, Brayne C (2005). Global prevalence of dementia: a delphi consensus study. *Lancet*.

[24] Grant WB (1997). Dietary links to Alzheimer’s disease. *Alzheimer’s Disease Review*.

[25] Huang X, Atwood CS, Hartshorn MA (1999). The A*β* peptide of Alzheimer’s disease directly produces hydrogen peroxide through metal ion reduction. *Biochemistry*.

[26] Nelson TJ, Alkon DL (2005). Oxidation of cholesterol by amyloid precursor protein and *β*-amyloid peptide. *Journal of Biological Chemistry*.

[27] Multhaup G, Schlicksupp A, Hesse L (1996). The amyloid precursor protein of Alzheimer’s disease in the reduction of copper(II) to copper(I). *Science*.

[28] White AR, Multhaup G, Galatis D (2002). Contrasting, species-dependent modulation of copper-mediated neurotoxicity by the Alzheimer’s disease amyloid precursor protein. *Journal of Neuroscience*.

[29] Sayre LM, Perry G, Harris PLR, Liu Y, Schubert KA, Smith MA (2000). In situ oxidative catalysis by neurofibrillary tangles and senile plaques in Alzheimer’s disease: a central role for bound transition metals. *Journal of Neurochemistry*.

[30] Ma Q, Li Y, Du J (2006). Copper binding properties of a tau peptide associated with Alzheimer’s disease studied by CD, NMR, and MALDI-TOF MS. *Peptides*.

[31] Hill GM, Brewer GJ, Prasad AS (1987). Treatment of Wilson’s disease with zinc. I. Oral zinc therapy regimens. *Hepatology*.

[32] Holden JM, Wolf WR, Mertz W (1979). Zinc and copper in self-selected diets. *Journal of the American Dietetic Association*.

[33] Klevay LM, Reck SJ, Barcome DF (1979). Evidence of dietary copper and zinc deficiencies. *Journal of the American Medical Association*.

[34] Reiser S, Smith JC, Mertz W (1985). Indices of copper status in humans consuming a typical American diet containing either fructose or starch. *American Journal of Clinical Nutrition*.

[35] Brewer GJ, Danzeisen R, Stern BR (2010). Letter to the editor and reply: toxicity of copper in drinking water. *Journal of Toxicology and Environmental Health*.

[36] Danzeisen R, Stern BR, Aggett PJ (2010). Reply to George Brewer letter to the editor: toxicity of copper in drinking water. *Journal of Toxicology and Environmental Health*.

[37] National Research Council (U.S.). Committee on Copper in Drinking Water (2000). *Copper in Drinking Water*.

[38] Kessler H, Bayer TA, Bach D (2008). Intake of copper has no effect on cognition in patients with mild Alzheimer’s disease: a pilot phase 2 clinical trial. *Journal of Neural Transmission*.

[39] Miyata M, Smith JD (1996). Apolipoprotein E allele-specific antioxidant activity and effects on cytotoxicity by oxidative insults and *β*-amyloid peptides. *Nature Genetics*.

[40] Moalem S, Percy ME, Andrews DF (2000). Are hereditary hemochromatosis mutations involved in Alzheimer disease?. *American Journal of Medical Genetics*.

[41] Zambenedetti P, De Bellis G, Biunno I, Musicco M, Zatta P (2003). Transferrin C2 variant does confer a risk for Alzheimer’s disease in caucasians. *Journal of Alzheimer’s Disease*.

[42] Seshadri S, Beiser A, Selhub J (2002). Plasma homocysteine as a risk factor for dementia and Alzheimer’s disease. *New England Journal of Medicine*.

[43] Nakano E, Williamson MP, Williams NH, Powers HJ (2004). Copper-mediated LDL oxidation by homocysteine and related compounds depends largely on copper ligation. *Biochimica et Biophysica Acta*.

[44] Crouch PJ, Lin WH, Adlard PA (2009). Increasing Cu bioavailability inhibits A*β* oligomers and tau phosphorylation. *Proceedings of the National Academy of Sciences of the United States of America*.

[45] Ueda K, Kawano H, Hasuo Y, Fujishima M (1992). Prevalence and etiology of dementia in a Japanese community. *Stroke*.

[46] White L, Petrovitch H, Ross GW (1996). Prevalence of dementia in older Japanese-American men in Hawaii: the Honolulu-Asia aging study. *Journal of the American Medical Association*.

[47] Hill GM, Brewer GJ, Juni JE (1986). Treatment of Wilson’s disease with zinc. II. Validation of oral 64coppper with copper balance. *American Journal of the Medical Sciences*.

[48] Nose Y, Kim BE, Thiele DJ (2006). Ctr1 drives intestinal copper absorption and is essential for growth, iron metabolism, and neonatal cardiac function. *Cell Metabolism*.

[49] Salustri C, Barbati G, Ghidoni R (2010). Is cognitive function linked to serum free copper levels? A cohort study in a normal population. *Clinical Neurophysiology*.

[50] Brewer GJ, Kanzer SH, Zimmerman EA, Celmins DF, Heckman SM, Dick R (2010). Copper and ceruloplasmin abnormalities in Alzheimers disease. *American Journal of Alzheimer’s Disease and other Dementias*.

[51] Arnal N, Cristalli DO, de Alaniz MJT, Marra CA (2010). Clinical utility of copper, ceruloplasmin, and metallothionein plasma determinations in human neurodegenerative patients and their first-degree relatives. *Brain Research*.

[52] Danzeisen R, Araya M, Harrison B (2007). How reliable and robust are current biomarkers for copper status?. *British Journal of Nutrition*.

[53] Brewer GJ, Dick RD, Johnson VD (1998). Treatment of Wilson’s disease with zinc: XV long-term follow-up studies. *The Journal of Laboratory and Clinical Medicine*.

[54] Kessler H, Pajonk FG, Meisser P (2006). Cerebrospinal fluid diagnostic markers correlate with lower plasma copper and ceruloplasmin in patients with Alzheimer’s disease. *Journal of Neural Transmission*.

[55] Bayer TA, Schäfer S, Simons A (2003). Dietary Cu stabilizes brain superoxide dismutase 1 activity and reduces amyloid A*β* production in APP23 transgenic mice. *Proceedings of the National Academy of Sciences of the United States of America*.

[56] Klevay LM (2008). Alzheimer’s disease as copper deficiency. *Medical Hypotheses*.

[57] Hedera P, Fink JK, Bockenstedt PL, Brewer GJ (2003). Myelopolyneuropathy and pancytopenia due to copper deficiency and high zinc levels of unknown origin: further support for existence of a new zinc overload syndrome. *Archives of Neurology*.

[58] Hedera P, Peltier A, Fink JK, Wilcock S, London Z, Brewer GJ (2009). Myelopolyneuropathy and pancytopenia due to copper deficiency and high zinc levels of unknown origin II. The denture cream is a primary source of excessive zinc. *NeuroToxicology*.

